# Editorial

**Published:** 2016-12-01

**Authors:** Prof Hasan Khan Kamrul

**Affiliations:** Vice Chancellor, Bangabandhu Sheikh Mujib Medical University, Dhaka, Bangladesh, E-mail: vc@bsmmu.edu.bd

Dear Colleagues,

It is my extreme pleasure to compile an editorial for the journal entitled “Euroasian Journal of Hepato-Gastroenterology.” It is needless to emphasis the importance of scientific journal for dissemination of knowledge, providing guidelines, and interconnecting civil and scientific societies. The importance of journals is further manifested in developing and resource-constrained countries. I also view the importance of medical journal from a novel perspective of globalization. In this evolving world, new and novel information and advanced techniques have been piling up in developed and advanced countries. On the contrary, developing countries have been harboring most of the patients of different specialties and subspecialties. In fact, many of these patients have not been properly diagnosed and managed. A bridge between these realities of developed/ advanced countries versus developing/resource-constrained nations may be only be addressed by compiling and circulating medical journals.

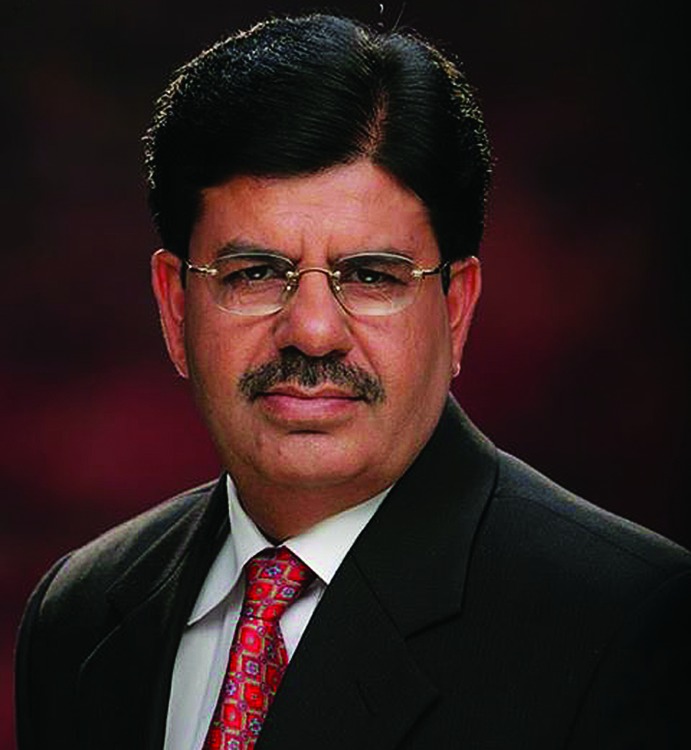



I am really excited to know that more than 10 countries of Europe and Asia are actively involved in designing and proper execution of regular publication of “Euroasian Journal of Hepato-Gastroenterology.” The journal has successfully completed its 6th birthday without any break or discontinuation. Medical scientists and clinicians of 25 countries enrolling continents of Asia, Africa, Europe, and America have sent their articles for this peer-reviewed journal.

At the top of these facts, I am especially excited to know the fact that the journal is compiled by combined efforts of hepato-gastroenterologists of Asia and Central Europe: it is published from India. It is a well-known fact that the medical scientists of Asia and Central Europe are lagging behind those of Europe, USA, and Japan. However, the majority population of the world is from these parts of the globe. The entire approach of this journal is to provide what is needed but remain absent for decades. The journal is indexed in several citation bodies and the editors and publishers are doing their best to incorporate the journal in PubMed. I hope that their dream would come into reality and we would soon find the existence of this journal in PubMed and acquisition of an impact factor may not be a remote goal.

I also feel proud that this journal has become an important publication pot for the researchers and clinicians of Bangabandhu Sheikh Mujib Medical Universities and Bangladeshi medical scientists of other institutions. Also, several medical scientists of Bangladesh have been working in the capacity of editors and reviewers for this journal.

Taken all facts into a comprehensive outlook, I would congratulate the initiators of the journal, the editorial board, and publishers and hoping for eternal existence of this booklet of science for our development and searching for identity.

**Prof Kamrul Hasan Khan**

Vice Chancellor

Bangabandhu Sheikh Mujib Medical University

Dhaka, Bangladesh

E-mail: vc@bsmmu.edu.bd

